# Microwave-assisted synthesis of novel Ti/BTB-MOFs as porous anticancer and antibacterial agents

**DOI:** 10.3389/fchem.2024.1386311

**Published:** 2024-05-13

**Authors:** Ali Altharawi, Safar M. Alqahtani, Taibah Aldakhil, Irfan Ahmad

**Affiliations:** ^1^ Department of Pharmaceutical Chemistry, College of Pharmacy, Prince Sattam Bin Abdulaziz University, Al-Kharj, Saudi Arabia; ^2^ Department of Clinical Laboratory Sciences, College of Applied Medical Science, King Khalid University, Abha, Saudi Arabia

**Keywords:** titanium-BTB metal-organic framework, anticancer agents, bone cancer cells, skin cancer cells, antibacterial agents

## Abstract

Nano compounds, especially metal-organic frameworks (MOFs), have significant properties. Among the most important properties of these compounds, which depend on their specific surface area and porosity, are biological properties, such as anticancer and antibacterial properties. In this study, a new titanium/BTB metal-organic framework (Ti/BTB-MOF) was synthesized by using titanium nitrate and 1,3,5-Tris(4-carboxyphenyl)benzene (BTB) under microwave radiation. The structure of the synthesized Ti/BTB-MOF was characterized and confirmed using X-ray diffraction (XRD) patterns, X-ray photoelectron spectroscopy (XPS) analysis, Fourier transform infrared (FT-IR) spectra, energy-dispersive X-ray (EDAX) analysis mapping, scanning electron microscope (SEM) images, thermogravimetric analysis (TGA) curves, and Brunauer–Emmett–Teller (BET) analysis. The *in vitro* anticancer properties of Ti/BTB-MOF were evaluated using the MTT method against MG-63/bone cancer cells and A-431/skin cancer cells. The *in vitro* antibacterial activity was tested using the Clinical and Laboratory Standards Institute (CLSI) guidelines. In the anticancer activity, IC_50_ (half-maximal inhibitory concentration) values of 152 μg/mL and 201 μg/mL for MG-63/bone cancer cells and A-431/skin cancer cells, respectively, were observed. In the antibacterial activity, minimum inhibitory concentrations (MICs) of 2–64 μg/mL were observed against studied pathogenic strains. The antimicrobial activity of Ti/BTB-MOF was higher than that of penicillin and gentamicin. Therefore, the synthesized Ti/BTB-MOF could be introduced as a suitable bioactive candidate.

## 1 Introduction

Cancer is a common cause of human mortality (Turner et al., 2020). Although methods such as chemotherapy and laser are developing in the treatment of cancer, the reporting of compounds and the presence of new compounds with anticancer properties are still important (Dallavalle et al., 2020; Anduran et al., 2022; Melfi et al., 2023).

Bacteria, a common cause of disease in humans, can also cause the death of humans (Hussain et al., 2022). The excessive use of antibiotics to suppress these pathogenic agents has led to the resistance of some strains (Şen Karaman et al., 2020; Murugaiyan et al., 2022). Providing new and innovative antibiotics is one way to deal with this problem (Terreni et al., 2021).

**FIGURE 1:**
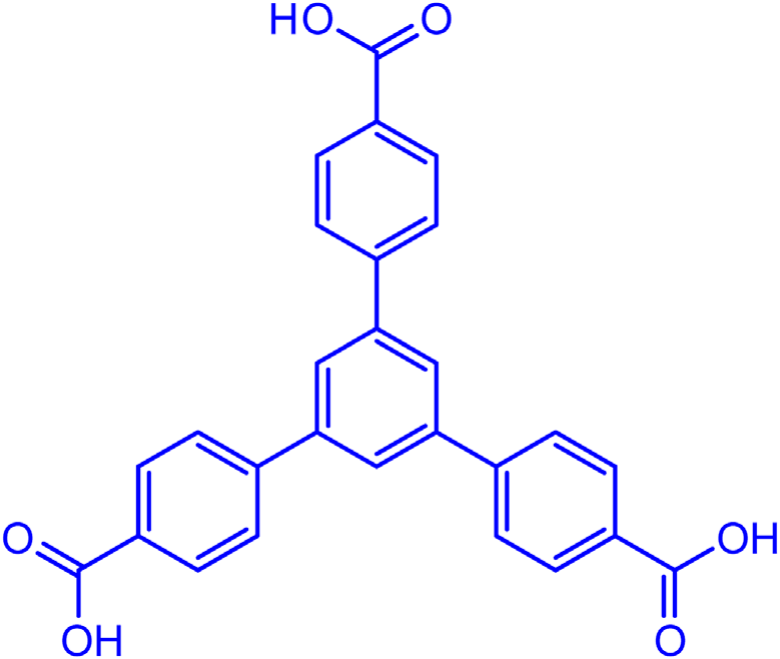
Structure of 1,3,5-Tris(4-carboxyphenyl)benzene.

Nanotechnology and metal-organic frameworks (MOFs) have found a worthy place in medical science (Bieniek et al., 2021; de Alencar Filho et al., 2021). In MOFs, which are composed of ligands and metals, the most important roles of ligands are flexibility and the possibility of controlling the size and environment of the holes (Cai et al., 2021; Shen et al., 2021; Kumar et al., 2023). The properties of the ligand can be preserved in the final product and give the final product the properties of the ligand (Joyce et al., 2021; Sharifzadeh et al., 2021). 1,3,5-Tris(4-carboxyphenyl)benzene (Figure 1) is an organic compound consisting of four benzene rings and three carboxylic acid groups.

Some biological properties such as anticancer activity (Wani and Zargar, 2023) have been reported from 1,3,5-tris(4-carboxyphenyl)benzene, which is also called BTB. Because it has three carboxylic acid groups in its structure, BTB can coordinate with metals and create MOFs. Several metals, such as Cu (Ji et al., 2023), Fe (Viltres et al., 2024), and Zn (Choi et al., 2023) have been reported to synthesize with BTB to create MOFs. These MOFs have been used as electrochemical sensors (Ji et al., 2023), to eliminate antibiotics from water (Viltres et al., 2024), in the adsorption of cationic dyes (Li et al., 2021), in hydrocarbon adsorption/separation (Wang et al., 2020), etc.

Another main component of MOFs is the metal. Metals, like ligands, can maintain their properties in MOF compounds (Qiu et al., 2020; Seidi et al., 2020; He et al., 2021). The d-block elements of the periodic table have a high ability as metals in the synthesis of MOF compounds (Roslan and Aris, 2023). Titanium, whose position is in Group 4 and d-block elements of the periodic table, has been used as a catalyst in the synthesis of chemicals (Samhitha et al., 2022) and as an antibacterial (Aslam et al., 2021), anticancer (Samhitha et al., 2022), and antioxidant agent (Muthuvel et al., 2021).

In this study, a microwave-assisted method was used to synthesize Ti/BTB-MOF nanostructures. This route is fast, controllable, and economical compared to other methods. In addition, this effective method has produced Ti/BTB-MOF samples with favorable physicochemical properties, which have affected the antibacterial applications of the final compound. Another advantage of this study is the synthesis of Ti/BTB-MOFs as novel anticancer and antibacterial candidates that can be used in other biological fields due to their practical properties.

## 2 Materials, methods, and characterization

### 2.1 Materials

The titanium (IV) nitrate (99.9%) was obtained from Sigma-Aldrich. The 1,3,5-Tris(4-carboxyphenyl)benzene (98%) was obtained from TCI Chemicals.

The bone cancer cells (MG-63), skin cancer cells (A-431), and the pathogenic bacterial strains studied were obtained from the American Type Culture Collection (ATCC).

### 2.2 Methods

#### 2.2.1 Titanium/BTB metal-organic framework (Ti/BTB-MOF)

Using ultrasonic, 1.4 mmol 1,3,5-benzene-tri-4-carboxyphenyl (BTB) was dispersed in 20 mL double distilled water. A-1 mmol aliquot of titanium (IV) nitrate was added to the mixture and stirred for 10 min (800 rpm) at room temperature. The mixture was subjected to microwave irradiation for 15 min (power of 350 W) (Bashar et al., 2022). The synthesized titanium/BTB-MOF was washed three times with EtOH and then three times with H_2_O. For drying, the synthesized Ti/BTB-MOF was placed under vacuum in an oven at 100°C for 3 h.

#### 2.2.2 Anticancer tests

The *in vitro* anticancer activity of Ti/BTB-MOF was investigated using MTT (3-[4,5-dimethylthiazol-2-yl]-2,5 diphenyl tetrazolium bromide) assay protocols (Rhodes, 1996; Moghaddam-manesh et al., 2021; Alkhatami et al., 2023). In the evaluation and tests, concentrations of 5 μg/mL, 10 μg/mL, 20 μg/mL, 40 μg/mL, 80 μg/mL, 160 μg/mL, and 320 μg/mL of titanium/BTB-MOF were prepared and treated separately with MG-63/bone cancer cells and A-431/skin cancer cells for 24 h and 48 h.

#### 2.2.3 Antibacterial tests

*In vitro* antibacterial tests of Ti/BTB-MOF were investigated using the Clinical and Laboratory Standards Institute (CLSI) and antimicrobial susceptibility testing methods (Etemadi et al., 2016; Moghaddam-Manesh et al., 2020; Terreni et al., 2021). The antibacterial activity of titanium/BTB-MOF was investigated against the pathogenic strains ATCC 33809 (*Vibrio fluvialis*), ATCC 25729 (*Rhodococcus equi*), ATCC 29178 (*Streptococcus iniae*), ATCC 9610 (*Yersinia enterocolitica*), ATCC 13313 (*Shigella dysenteriae*), and ATCC 19115 (*Listeria monocytogenes*).

### 2.3 Characterization and equipment

X-ray diffraction (XRD) patterns, X-ray photoelectron spectroscopy (XPS) analysis, Fourier transform infrared (FT-IR) spectra, energy-dispersive X-ray (EDAX analysis mapping, scanning electron microscope (SEM) images, thermogravimetric analysis (TGA) curves, and Brunauer–Emmett–Teller (BET) analysis were used to characterize and confirm the structure of Ti/BTB-MOF. The equipment used for analysis were a DW-XRD-Y3000 (XRD), a SPECS Phoibos 150 (UHV-XPS), a Thermo Nicolet Avatar 360 (FT-IR), a TESCAN MIRA3 (EDAX, EDAX mapping, and SEM), a TA Instruments SDT-Q600 (TGA), and a BELSORP mini II (BET).

In antibacterial activity tests, the required concentration of the studied strains was prepared using a Jenway 7315 UV/Visible spectrophotometer.

In anticancer activity tests, a KERN OCM 161 inverted microscope was used to count cancer cells, and an Accuris MR9610 SmartReader UV-Vis (115 V) was used to measure absorbance.

## 3 Results

### 3.1 Ti/BTB-MOF synthesis results

**FIGURE 2:**
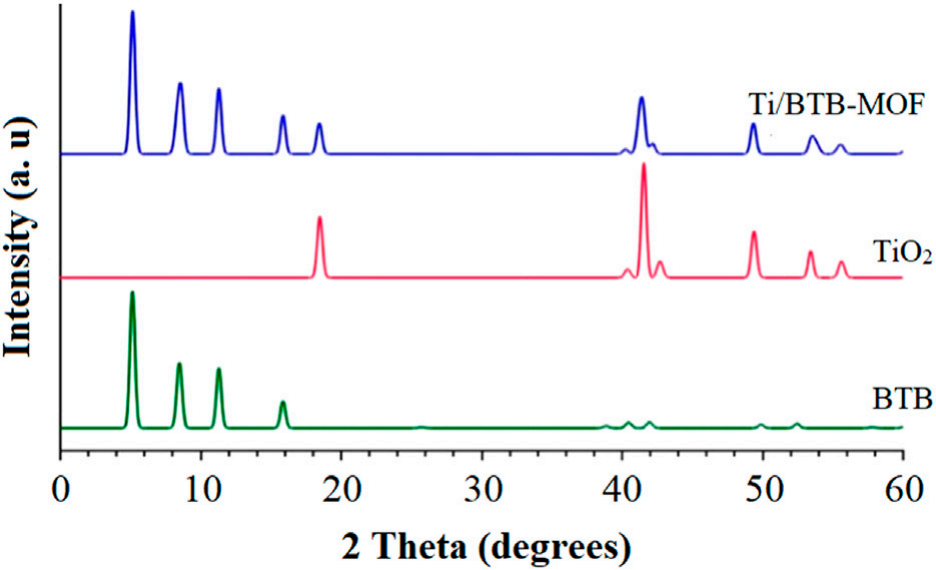
The XRD patterns of Ti/BTB-MOF.

The XRD pattern of Ti/BTB-MOF is given in Figure 2.

In the XRD pattern of Ti/BTB-MOF, plates [011], [002], [121], [222], and [132] were observed at 2Θ.

**FIGURE 3:**
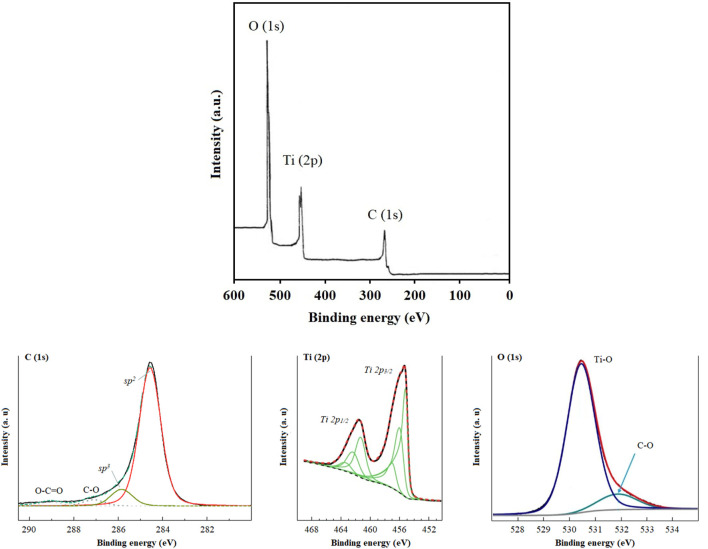
The XPS analysis of Ti/BTB-MOF.

In the XPS analysis of Ti/BTB-MOF (Figure 3), binding energies of 283 eV, 458 eV, 464 eV, and 532 eV were observed.

**FIGURE 4:**
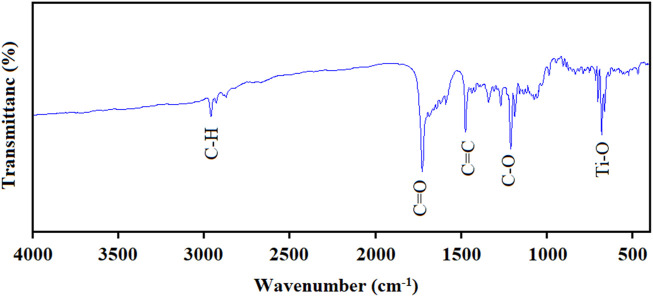
The FT-IR spectrums of Ti/BTB-MOF.

In the FT-IR spectrum related to the Ti/BTB-MOF, as shown in Figure 4, absorptions at 655 cm^−1^, 1,150 cm^−1^, 1,420 cm^−1^, 1710 cm^−1^, and 2,900 cm^−1^ were observed.

**FIGURE 5:**
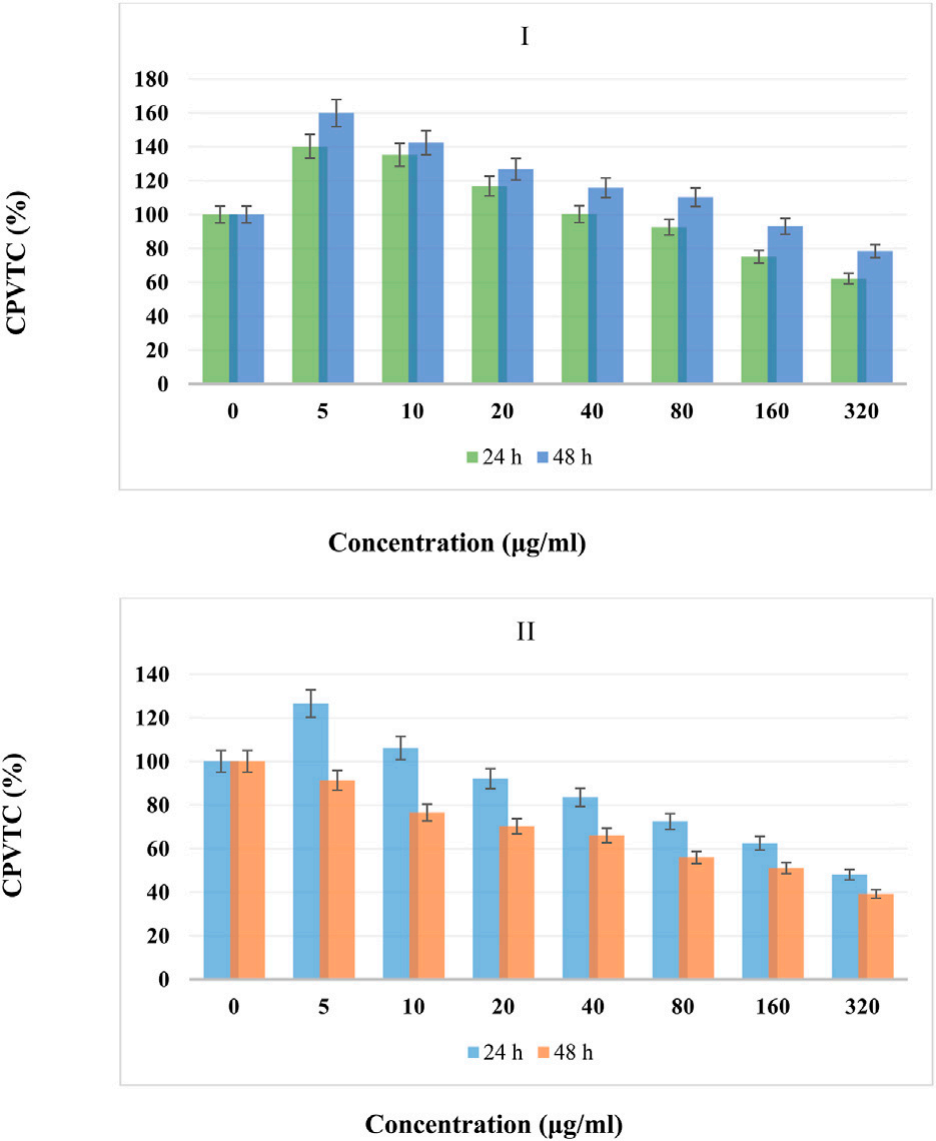
The CPVTC of BTB (I) and Ti/BTB-MOF (II) against skin cancer cells, (n = 3) ± SD.

Figure 5 shows the thermal stability of the final product. Two areas of basic weight loss were observed in the TGA curve in the regions of 350°C and 480°C.

**FIGURE 6:**
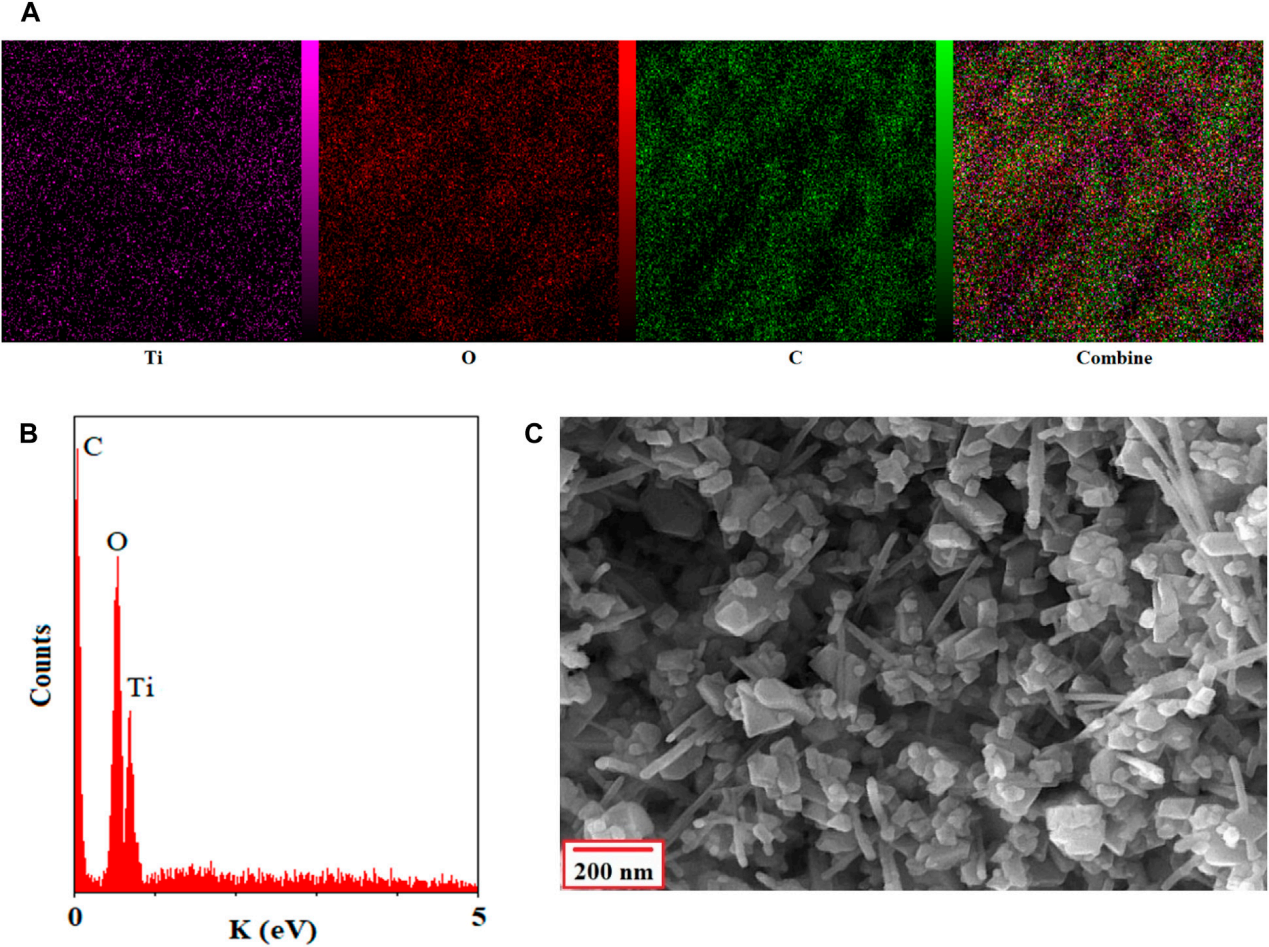
The EDAX **(A)**, EDAX mapping **(B)**, and SEM image **(C)** of Ti/BTB-MOF.

The nitrogen adsorption and desorption curves of Ti/BTB-MOF are shown in Figure 6.

The obtained BET, mean pore diameter, and Barrett–Joyner–Halenda (BJH) pore volume are given in Table 1.

**TABLE 1:** BET, Barrett–Joyner–Halenda (BJH) pore volume, and mean pore diameter of Ti/BTB-MOF.

BET (m^3^/g)	BJH pore volume (cm^3^/g)	Mean pore diameter (nm)
35	0.42	1.36

### 3.2 Anticancer test results

#### 3.2.1 Anti-bone cancer cells

**FIGURE 7:**
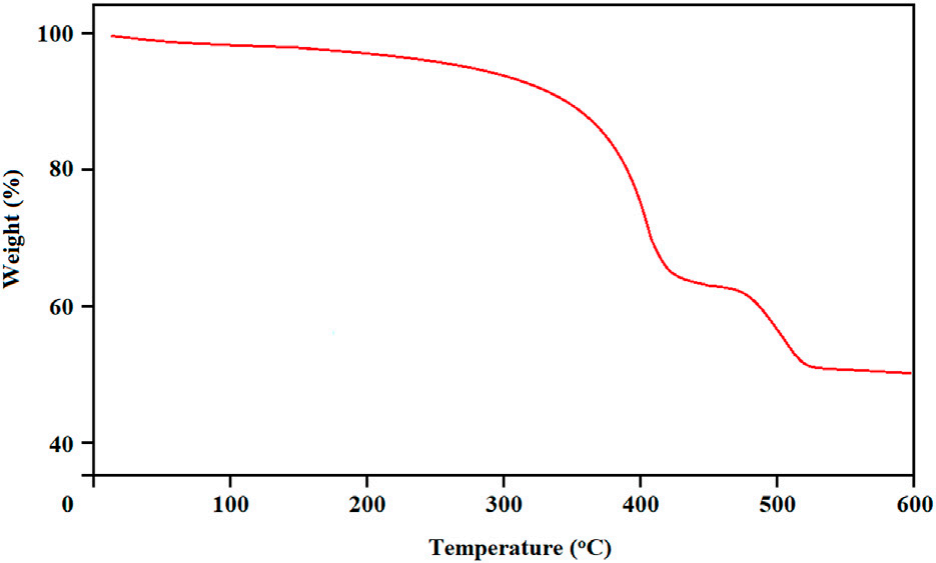
The TGA curve of Ti/BTB-MOF.

The cell proliferation and viability compared to control (CPVTC) of different concentrations of BTB (I) and Ti/BTB-MOF (II) against bone cancer cells at temperatures of 24 h and 48 h are given in Figure 7.

Based on the obtained results, at 24 h, the CPVTC for concentrations of 5 μg/mL, 10 μg/mL, 20 μg/mL, 40 μg/mL, 80 μg/mL, 160 μg/mL, and 320 μg/mL of Ti/BTB-MOF were obtained as 114%, 99% 85%, 73%, 62%, 54%, and 42% compared to the control, respectively.

At 48 h, the CPVTC for concentrations of 5 μg/mL, 10 μg/mL, 20 μg/mL, 40 μg/mL, 80 μg/mL, 160 μg/mL, and 320 μg/mL of Ti/BTB-MOF were obtained as 85%, 71%, 66%, 59%, 52%, 43%, and 27% compared to the control, respectively.

For BTB at 24 h, the CPVTC for concentrations of 5 μg/mL, 10 μg/mL, 20 μg/mL, 40 μg/mL, 80 μg/mL, 160 μg/mL, and 320 μg/mL were obtained as 167%, 150% 131%, 120%, 109%, 95%, and 81% compared to the control, respectively.

At 48 h, the CPVTC for concentrations of 5 μg/mL, 10 μg/mL, 20 μg/mL, 40 μg/mL, 80 μg/mL, 160 μg/mL, and 320 μg/mL of BTB were obtained as 155%, 142%, 127%, 103%, 99%, 80%, and 66% compared to the control, respectively.

#### 3.2.2 Anti-skin cancer cells

**FIGURE 8:**
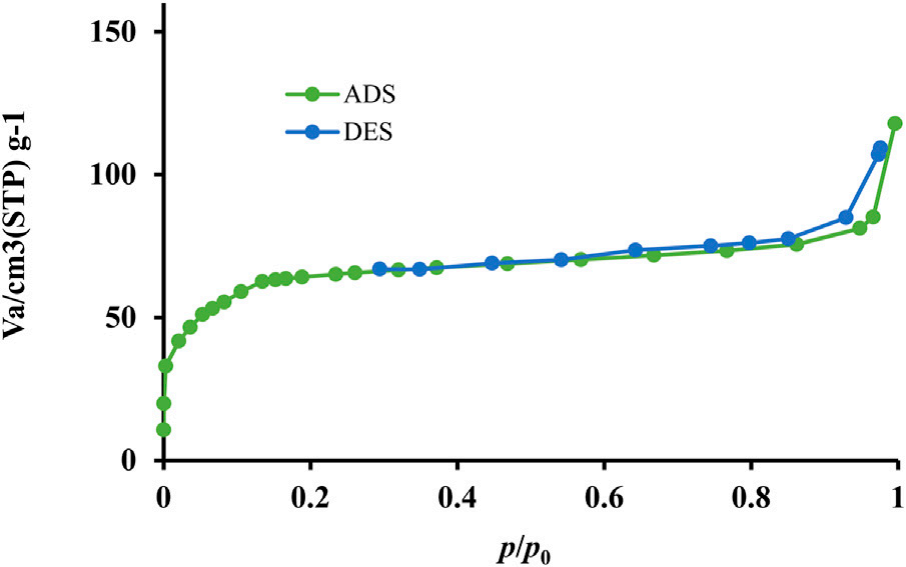
The nitrogen adsorption and desorption curves of Ti/BTB-MOF.

The CPVTC of different concentrations of BTB (I) and Ti/BTB-MOF (II) against skin cancer cells at 24 h and 48 h are given in Figure 8.

Based on the obtained results, at 24 h, the CPVTC for concentrations of 5 μg/mL, 10 μg/mL, 20 μg/mL, 40 μg/mL, 80 μg/mL, 160 μg/mL, and 320 μg/mL of Ti/BTB-MOF were obtained as 126%, 106% 92%, 83%, 72%, 62%, and 48% compared to the control, respectively.

At 48 h, the CPVTC for concentrations of 5 μg/mL, 10 μg/mL, 20 μg/mL, 40 μg/mL, 80 μg/mL, 160 μg/mL, and 320 μg/mL of Ti/BTB-MOF were obtained as 91%, 76%, 70%, 65%, 55%, 51%, and 39% compared to the control, respectively.

For BTB at 24 h, the CPVTC for concentrations of 5 μg/mL, 10 μg/mL, 20 μg/mL, 40 μg/mL, 80 μg/mL, 160 μg/mL, and 320 μg/mL were obtained as were obtained as 159%, 142% 126%, 115%, 110%, 93%, and 78% compared to the control, respectively.

At 48 h, the CPVTC for concentrations of 5 μg/mL, 10 μg/mL, 20 μg/mL, 40 μg/mL, 80 μg/mL, 160 μg/mL, and 320 μg/mL of BTB were obtained as 140%, 135%, 116%, 100%, 92%, 75%, and 62% compared to the control, respectively.

#### 3.2.3 Antibacterial test results

The antibacterial activities of BTB, Ti/BTB-MOF, penicillin, and gentamicin were investigated. The minimum inhibitory concentration (MIC), minimum bactericidal concentration (MBC), and inhibitory zone diameter (IZD) were tested and reported. The tests were repeated three times, and the results, which are the average of three repetitions, are shown in Table 2.

**TABLE 2:** Antibacterial activity of Ti/BTB-MOF (mean, n = 3).

Compound strain	BTB			Ti/BTB-MOF			Penicillin			Gentamicin		
	MIC μg/mL	MBC μg/mL	IZD mm	MIC μg/mL	MBC μg/mL	IZD mm	MIC μg/mL	MBC μg/mL	IZD mm	MIC μg/mL	MBC μg/mL	IZD mm
ATCC 33809	-	-	-	64	128	15.37	-	-	-	-	-	-
ATCC 25729	64	128	17.01	16	32	17.93	4	8	19.63	2	4	20.42
ATCC 29178	128	256	13.25	2	4	20.64	2	4	18.75	4	16	18.31
ATCC 9610	-	-	-	32	64	14.92	-	-	-	-	-	-
ATCC 13313	16	32	16.71	4	16	19.31	32	64	15.27	2	4	22.43
ATCC 19115	32	64	19.49	2	4	20.08	1	2	20.75	-	-	-

## 4 Discussion

### 4.1 Synthesis of Ti/BTB-MOF

In this study, a novel Ti/BTB-MOF was synthesized using titanium (IV) nitrate and 1,3,5-Tris(4-carboxyphenyl)benzene under microwave irradiation. The purpose of using the microwave method is to provide optimal conditions for forming MOF nanostructures with desirable properties such as small particle size distribution, desirable thermal stability, and good textural properties. The mechanism of formation of MOF nanostructures by using a microwave-assisted method is developed in this study based on previous literature (Choi et al., 2008; Couzon et al., 2022; Fernández-Andrade et al., 2023).

The most important factor in the formation of products using the microwave-assisted method is the heating process. The basis of this process is the interaction between the electrical component of microwaves and polar compounds. Due to the unique characteristics of these waves when heating during chemical reactions, microwaves have been widely used to synthesize nanomaterials, MOFs, organic compounds, etc. In this study, microwave irradiation with a power of 350 W was used as the reaction condition. A literature review showed that a power of 350 W leads to a temperature close to 130°C (Xiaokang et al., 2020).

The structure of the final product was predicted and confirmed using the XRD patterns, XPS analysis, EDAX analysis, and EDAX mapping. Then, other characteristics of nanoparticles were investigated and studied using the SEM images, TGA curves, and the BET technique.

The XRD pattern of the final product (Figure 2) was similar to the XRD pattern reported for the crystal structure of titanium nanoparticles (Gómez-Avilés et al., 2020). The XRD patterns of the samples indicate that the nanostructures developed in this study have a higher percentage of crystalline phases than previous reports (Gómez-Avilés et al., 2020). This difference can be related to the efficient effects of the microwave-assisted route on the crystalline properties of the final product.

Using XRD data and the Debye–Scherrer equation, the synthesized Ti/BTB-MOF was 68 nm (Holzwarth and Gibson, 2011).

In the XPS analysis of Ti/BTB-MOF (Figure 3), the binding energies related to carbon (1s-283 eV) (Oh et al., 2013; Susi et al., 2015; Tudino et al., 2020), titanium (2p_1/2_–458 eV, and 2p_3/2_–464 eV) (Shvab et al., 2017), and oxygen (1s-532 eV) (Tudino et al., 2020) were observed.

The absorptions observed in the FT-IR spectrum (Figure 4) near 655 cm^−1^, 1,150 cm^−1^, 1,420 cm^−1^, 1710 cm^−1^, and 2,900 cm^−1^ were related to Ti-O (Al-Amin et al., 2016), C-O (Alkhatami et al., 2023), C=C (Alkhatami et al., 2023), C=O (Akhavan-Sigari et al., 2022), and C-H (Ahmad et al., 2022), respectively. The BTB has three carboxylic acid groups, but the broad band due to O-H groups (near 3,200–3,500 cm−1) was not observed in the spectrum of the final product.

**FIGURE 9:**
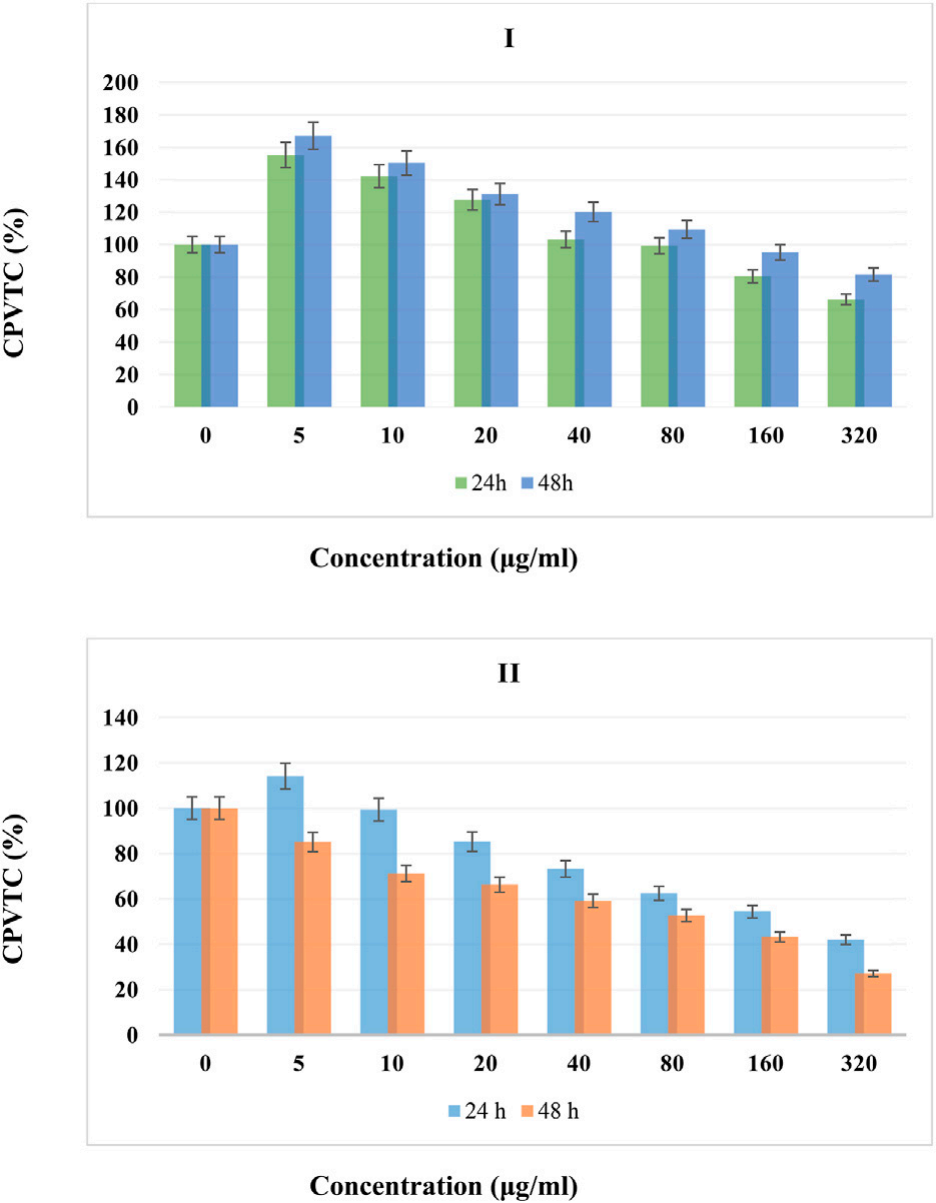
The CPVTC of BTB (I) and Ti/BTB-MOF (II) against bone cancer cells, (n = 3) ± SD.

Based on the EDAX and EDAX mapping of Ti/BTB-MOF (Figure 9A,B), titanium, carbon, and oxygen were observed in the structure of the final product.

**FIGURE 10:**
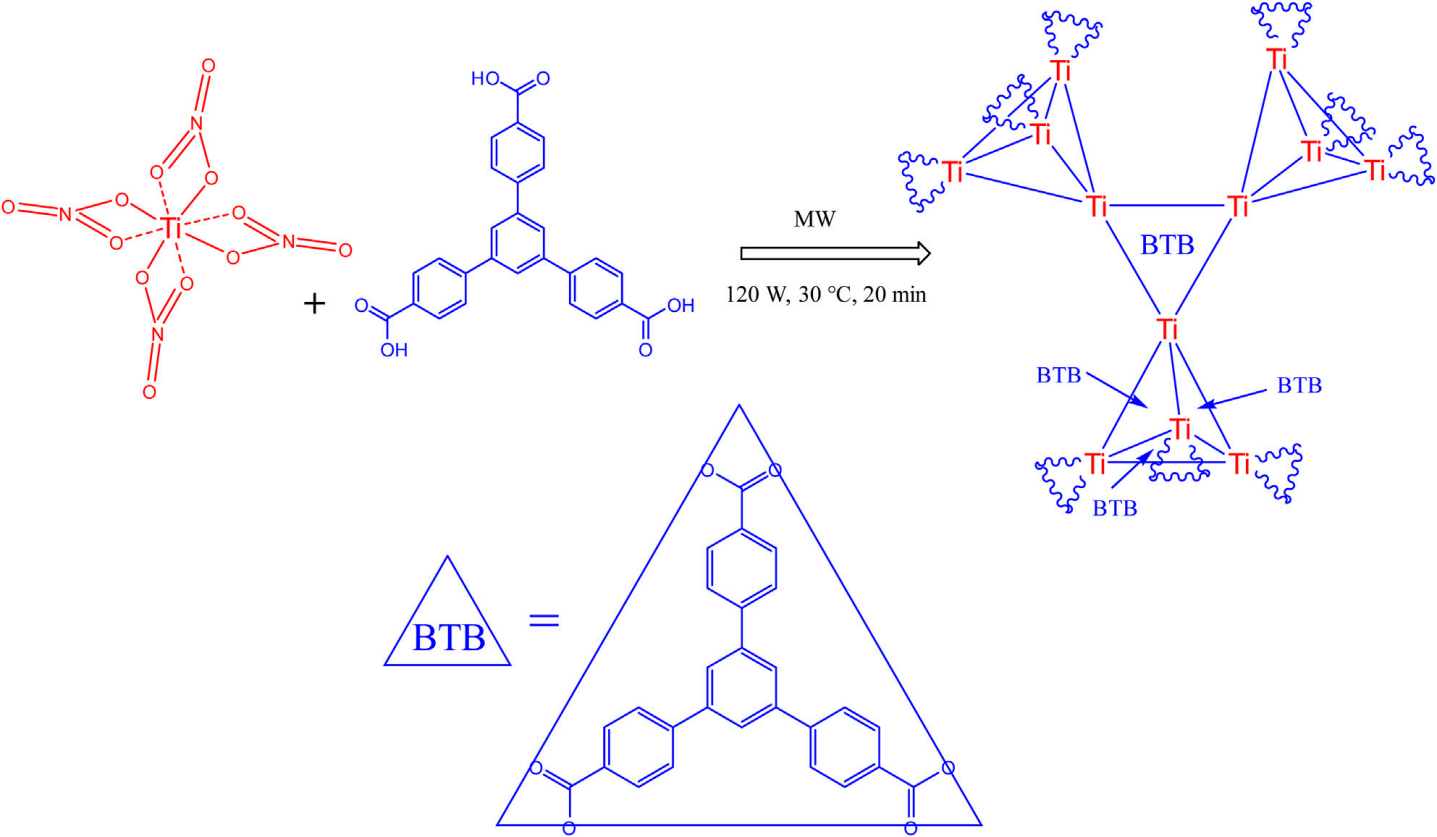
Proposed structure of the synthesized Ti/BTB-MOF.

Therefore, the structure of Figure 10 can be predicted for the novel Ti/BTB-MOF by using XRD patterns, which indicate the presence of titanium nanoparticles in Ti/BTB-MOF structure, the XPS analysis, which indicates the binding energies of carbon, titanium, and oxygen in the final product, FT-IR spectrum, which indicates the absorption of the elements of the raw materials and the bonding of Ti-O and the absence of O-H in the product, and EDAX, EDAX mapping, which proves the presence of carbon, titanium, and oxygen in the final product.

SEM, TGA, and BET were used to obtain other characteristics of the Ti/BTB-MOF.

The SEM image of the final product proves its uniformity (Figure 9C) and its nano-size. Uniform morphology and nano-size are parameters that depend on the synthesis method of nanoparticles (Yaqoob et al., 2020). Therefore, it can be concluded that the technique of synthesizing Ti/BTB-MOF using microwave radiation is suitable for this study.

Based on Figure 5, the weight loss observed at 350°C can be attributed to the destruction of BTB, and the weight loss observed at 480°C can be attributed to the destruction of the complex.

Using nitrogen adsorption and desorption curves (Figure 6) and the BET technique, the specific surface area for Ti/BTB-MOF was obtained as 35 m^2^/g. In nanoparticles, the specific surface area depends on the synthesizing technique (Sajid and Płotka-Wasylka, 2020). The high specific surface area indicates that this study used an appropriate synthesis method.

### 4.2 Anticancer activity of Ti/BTB-MOF

Figures 7, 8 show the best effectiveness and lowest CPVTC anticancer activity against bone and skin cancer cells at 48 h and 320 μg/mL of Ti/BTB-MOF.

**FIGURE 11:**
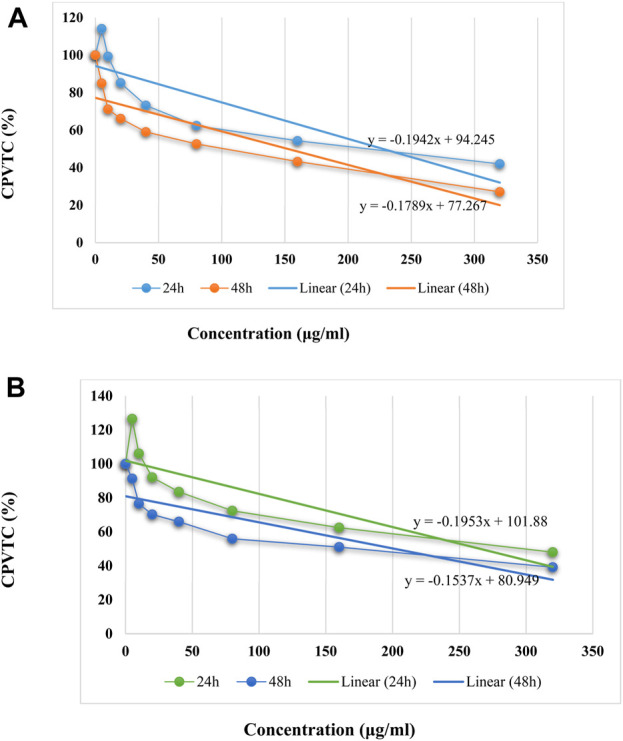
The linear equation of concentration and CPVTC curves for **(A)** bone cancer cells and **(B)** skin cancer cells, (n = 3) ± SD.

In anticancer activity, the IC_50_ values of Ti/BTB-MOF against bone cancer cells and skin cancer cells were calculated by using the linear equation of cell proliferation and viability and concentration curve (Figure 11). The IC_50_ values against bone cancer cells were 228 μg/mL (24 h) and 152 μg/mL (48 h), and the IC_50_ values against skin cancer cells were 266 μg/mL (24 h) and 201 μg/mL (48 h).

For BTB, the IC_50_ values against bone cancer cells were 453.73 μg/mL (24 h) and 354 μg/mL (48 h), and the IC_50_ values against skin cancer cells were 448 μg/mL (24 h) and 337 μg/mL (48 h).

Comparing the anticancer results of BTB and Ti/BTB-MOF indicated that the anticancer activity of Ti/BTB-MOF was higher than that of BTB. The reason can be attributed to the nanostructure that is formed and the presence of titanium in the final product.

Based on previous results, the IC_50_ values for doxorubicin, which is known as the standard drug against bone and skin cancer cells, have been reported to be close to 50 μg/mL (Rezadoost et al., 2019), which is about 1/3 of the values observed for Ti/BTB-MOF. However, because Ti/BTB-MOF has additional properties, such as its antibacterial activities, the synthesized compound is useful and important.

In the anticancer activities of Ti/BTB-MOF, the *p*-values of the IC_50_ values at 24 h and 48 h were calculated, and the results are given in Table 3.

**TABLE 3:** The *p*-values of the IC_50_ values of Ti/BTB-MOF against bone cancer cells and skin cancer cells.

Cancer cell	*p*-value 24 h (μg/mL)	*p*-value 48 h (μg/mL)
Bone cancer cells	0.002	0.000
Skin cancer cells	0.001	0.001

The *p*-value results of the investigation of anticancer activity against bone and skin cancer cells indicated that concentrations were a critical parameter at 24 and 48 h. By increasing the time and increasing the nanoparticle concentration, the contact of cancer cells with the Ti/BTB-MOF increases and leads to an increase in its anticancer properties. As previous studies have suggested, increasing the specific surface area increases biological activities (Cui and Zhu, 2021), so here, too, the high specific surface area of Ti/BTB-MOF leads to an increase in contact with cancer cells and increased anticancer properties. Other factors, such as the biological properties of the BTB (Wani and Zargar, 2023) and Ti (Aslam et al., 2021; Samhitha et al., 2022) in the Ti/BTB-MOF structure also influence the anticancer activity.

### 4.3 Antibacterial activity of Ti/BTB-MOF

In antibacterial evaluations, as shown in Table 2, MIC values against *V. fluvialis*, *R. equi*, *S. iniae*, *Y. enterocolitica*, *S. dysenteriae*, and *L. monocytogenes* were obtained as 128 μg/mL, 32 μg/mL, 4 μg/mL, 64 μg/mL, 16 μg/mL, and 4 μg/mL, respectively. The Ti/BTB-MOF against *S. iniae* had the highest effectiveness. Tests were performed on penicillin and gentamicin, well-known antibiotics on the market. The results showed that penicillin and gentamicin are ineffective on *V. fluvialis* and *Y. enterocolitica*, but significant effectiveness of Ti/BTB-MOF was observed, which is another unique capability of synthesized Ti/BTB-MOF. The antibacterial results of BTB against the studied strains were also higher than those of gentamicin in some strains, such as *L. monocytogenes*. However, the BTB results were generally lower than Ti/BTB-MOF, which can be attributed to the formed nanostructure and the presence of titanium.

The high antimicrobial properties of nanoparticles against the studied strains can also be due to their high specific surface area, leading to more contact and, therefore, more destruction (Khezerlou et al., 2018; Cui and Zhu, 2021). In addition, other factors, such as the biological properties of the BTB (Wani and Zargar, 2023) and Ti (Aslam et al., 2021; Samhitha et al., 2022) in Ti/BTB-MOF structure, also influence anticancer activity.

## 5 Conclusion

By using microwave radiation, novel titanium/1,3,5-Tris(4-carboxyphenyl)benzene metal-organic frameworks (Ti/BTB-MOFs) were synthesized. The structure of synthesized Ti/BTB-MOF was predicted using XRD, XPS, FT-IR, EDAX, and EDAX mapping. The Ti/BTB-MOF was characterized using SEM, TGA, and BET. The Ti/BTB-MOF had thermal stability up to 350°C. The specific surface area of Ti/BTB-MOF was 35 m^2^/g. The 68-nm particle size was another feature of the Ti/BTB-MOF. These characteristics proved that the use of microwave radiation was suitable for the synthesis of Ti/BTB-MOF. The anticancer properties of nanoparticles against bone and skin cancer cells were evaluated. In the investigation, IC_50_ values for bone and skin cancer cells were obtained as 152 μg/mL and 201 μg/mL, respectively. The antibacterial properties of the synthesized Ti/BTB-MOF were also evaluated, and MIC, MBC, and IZD were reported. The antibacterial activity of Ti/BTB-MOF was compared with trade drugs, and on some of the studied strains, Ti/BTB-MOF had better inhibition than the drugs.

## Data Availability

The original contributions presented in the study are included in the article/Supplementary Material; further inquiries can be directed to the corresponding author.
